# Bioengineering platforms for cell therapeutics derived from pluripotent and direct reprogramming

**DOI:** 10.1063/5.0040621

**Published:** 2021-07-06

**Authors:** Yoonhee Jin, Seung-Woo Cho

**Affiliations:** 1 Department of Biotechnology, Yonsei University, Seoul 03722, Republic of Korea; 2 Center for Nanomedicine, Institute for Basic Science (IBS), Seoul 03722, Republic of Korea; 3 Graduate Program of Nano Biomedical Engineering (NanoBME), Advanced Science Institute, Yonsei University, Seoul 03722, Republic of Korea

## Abstract

Pluripotent and direct reprogramming technologies hold great potential for tissue repair and restoration of tissue and organ function. The implementation of induced pluripotent stem cells and directly reprogrammed cells in biomedical research has resulted in a significant leap forward in the highly promising area of regenerative medicine. While these therapeutic strategies are promising, there are several obstacles to overcome prior to the introduction of these therapies into clinical settings. Bioengineering technologies, such as biomaterials, bioprinting, microfluidic devices, and biostimulatory systems, can enhance cell viability, differentiation, and function, in turn the efficacy of cell therapeutics generated via pluripotent and direct reprogramming. Therefore, cellular reprogramming technologies, in combination with tissue-engineering platforms, are poised to overcome current bottlenecks associated with cell-based therapies and create new ways of producing engineered tissue substitutes.

## I. INTRODUCTION

In recent years, advances have been made in pluripotent reprogramming and direct cellular reprogramming technologies; in this regard, researchers are continuing to develop novel cell therapies that can provide cures for many devastating diseases.^1^ The rapid expansion of cellular reprogramming research over the past decade has uncovered methods that utilize patient-derived cells to form mature cell types.^2^ The advent of cellular reprogramming technology allows researchers to harvest somatic cells from a patient's skin biopsy, the urine, or blood samples and reprogram these cells into induced pluripotent stem cells (iPSCs) or directly reprogram them into a desired cell type. These pluripotent reprogramming or direct reprogramming techniques may provide possible solutions to achieve the personalized medicine. The iPSCs and directly reprogrammed cells derived from the patients can be used to repair injured tissues or as diagnostic tools to screen for drugs or aid decision making with regard to the choice of appropriate treatment options. Despite substantial advances in our understanding of cellular reprogramming, there are still a number of hurdles that hinder the therapeutic prospects and widespread clinical use of iPSC-derived and directly reprogrammed cell therapies.^3,4^

**FIG. 1.**
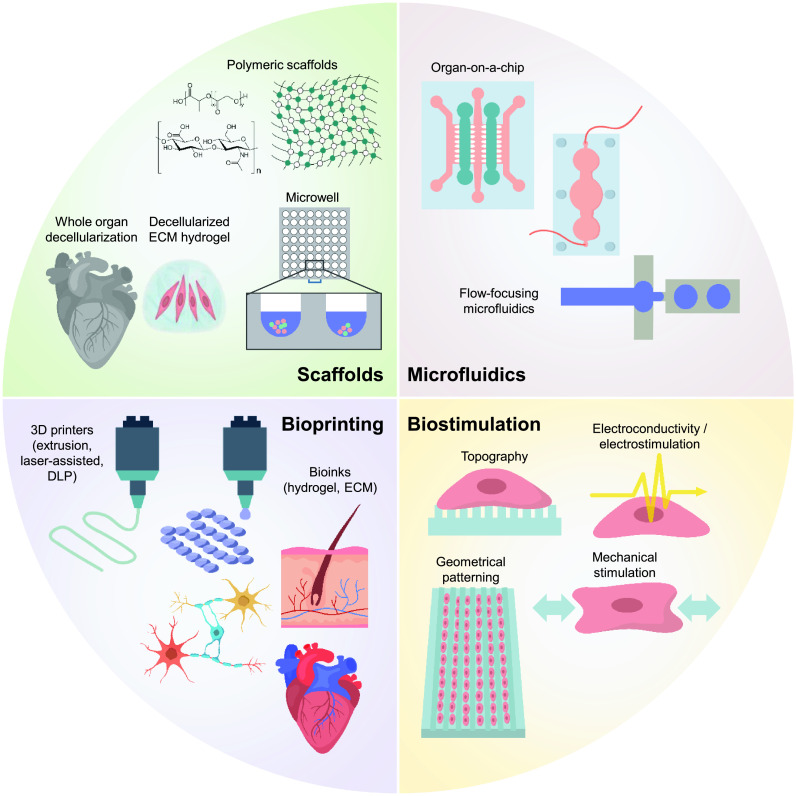
Bioengineering strategies to advance reprogrammed cell-based research. Promising technologies for improving the therapeutic efficacy and utility of the reprogrammed cells include biomaterial scaffolds, bioprinting, microfabricated devices, and biostimulatory systems.

Current obstacles to the clinical translation of reprogrammed cell derivatives include low reprogramming and differentiation efficiencies, low cell maturation and functionality, inefficient control of the cell state both pre- and post-transplantation, and low survival rates of cells during and after the engraftment *in vivo*. Careful design of cell cultivation systems and transplantation methods with bioengineering approaches can offer solutions to overcome these current limitations of reprogrammed cell therapies by modulating various cellular behaviors and functions. In this review, we will describe how bioengineering tools including biomaterials, microfluidics, bioprinting, and stimulatory devices can contribute to the development of novel therapeutic strategies with iPSC-derived and directly reprogrammed cells (Fig. 1).

## II. BIOMATERIALS

Moving from conventional two-dimensional (2D) to three-dimensional (3D) cultures could allow for the production of more physiologically relevant *in vitro* models of native tissue.^5,6^ The 3D culture of iPSC-cardiomyocytes (CMs) as aggregates^7,8^ or 3D multilayered sheets^9^ resulted in enhanced phenotypical, functional, and metabolic maturation compared to 2D monolayered cultures. Matrigel has often been used as the primary matrix for iPSC- and directly reprogrammed cell-based methodologies,^10^ but it is not a desirable material for clinical practices because of the unidentified xenogeneic components from mouse sarcoma cells and batch-to-batch variation that may lead to significant discrepancy in experimental outcomes.^11^ Along this line, natural and synthetic materials have been developed to create *in vivo*-like niches providing key elements to control the regulation of cell fate and function (Table I). With a well-designed configuration of matrix properties, functional scaffolds can regulate cell fate and trigger lineage-specific differentiation. Biochemical composition, extracellular matrix (ECM) stiffness, and degradation, as well as soluble factor signaling and cell–cell contact to physiologically relevant conditions, can be controlled.^5^ Furthermore, scaffolds can be designed as a cell delivery system that augments localization, retention, and survival of cells at the desired site.

**TABLE I.** Biomaterials for reprogrammed cell culture.

Biomaterials			Cells/constructs
Natural biomaterials	Protein-based	Collagen	3D skin construct^70^
			3D heart construct^100^
		Fibrin	Myogenic cells^15^
		Gelatin	Undifferentiated iPSCs^61^
			3D heart construct^63^
	Polysaccharide-based	Hyaluronic acid	Neural cells^21^
			Hepatocytes^68^
		Alginate	Undifferentiated iPSCs^50,55^
			Skin construct^70^
			3D heart construct^14^
		Chitosan	Undifferentiated iPSCs^52^
		Agarose	Undifferentiated iPSCs^52^
	Tissue-derived	Decellularized organ	Heart^26–29^
			Liver^30,31,33,34^
			Pancreas^35^
			Lungs^32,33^
			Kidneys^37,38^
		Decellularized extracellular matrix	Neural cells^41^
			Hepatocytes^41^
			Myogenic cells^76^
Synthetic biomaterials		Poly(ethylene glycol) (PEG)	Undifferentiated iPSCs^45^
		Polycaprolactone (PCL)	Neural cells^43^
		Poly(lactide-co-glycolide) (PLGA)	Brain organoids^46^
		Poly(N-isopropylacrylamide) (PNIPAM)	Endothelial cells^47^

### A. Natural scaffolds

Components of the naturally derived ECM, including fibrin,^12–15^ gelatin,^16–18^ hyaluronic acid,^19–21^ or a combination of these materials,^22^ have been used effectively to create 3D matrices to support iPSCs and directly reprogrammed cells. For the interest of directing differentiation, the scaffolds are often designed to mimic the ECM composition of the target tissue. For example, scaffolds for neurogenesis have been fabricated using hyaluronic acid,^19–21^ an abundant glycosaminoglycan found in the brain. Similarly, scaffolds for osteogenesis often contain hydroxyapatite,^23^ an inorganic mineral unique to bone tissue. Zhang *et al.* reported that 3D culture of human iPSC-derived neural progenitors (NPCs) in hyaluronic acid hydrogel promoted neuronal differentiation and maturation of NPCs compared to 2D culture.^20^ Wu *et al.* reported that human iPSC-NPCs underwent higher levels of neuronal differentiation in methacrylate-modified hyaluronic acid hydrogel with lower stiffness (≈0.51 kPa) than in more rigid hydrogel (≈1.41 kPa).^19^

Both collagen and fibrin hydrogels have been frequently used for a variety of tissue engineering applications because of their superior biocompatibility. Muller *et al.* built an engineered skin construct by seeding sensory neurons and Schwann cells derived from iPSCs at the bottom of a 3D fibroblast-populated collagen sponge.^24^ In comparison to 2D culture, this system enabled prolonged neuronal culture, which allowed cells to reach a further maturation stage. iPSC-sensory neurons formed a 3D nerve network spanning the whole construct only when co-cultured with iPSC-derived Schwann cells. In another study, Maffioletti *et al.* fabricated a 3D artificial skeletal muscle construct with multiple lineage cells using iPSC-derived myogenic cells, vascular endothelial cells (ECs), pericytes, and motor neurons.^15^ Myogenic differentiation in the construct was induced under continuous tension to the fibrin hydrogel. When the construct was generated using iPSC lines derived from patient with congenital muscular dystrophies, key pathological hallmarks were recapitulated, indicating that this platform could be used for disease modeling and drug testing.^15^

### B. Whole decellularized matrices

Another popular approach for producing scaffolds that recapitulate the tissue-specific microenvironment is decellularization. Whole organs have been decellularized to maintain the composition, biomechanical properties, microstructure, and shape of the native organs.^25^ Lu *et al.* for the first time generated bioartificial human hearts by repopulating whole decellularized mouse heart with human iPSC-derived cardiovascular progenitor cells via perfusion method.^26^ Seeded iPSC-cardiovascular progenitor cells differentiated *in situ* into CMs, ECs, and smooth muscle cells (SMCs). At 20 days of culture in the continuous perfusion system, the construct formed myocardium with vascular structures, spontaneous contraction, intracellular Ca^2+^ transients, and response to drugs. Scaling up this technique is imperative for the translation of these findings to the clinic. To this end, human hearts have been recellularized with iPSC-CMs to repopulate the parenchyma.^27^ After 4 to 7 days of cell seeding, the construct formed mature myocardial tissue with increased force production, electrical conductivity, left ventricular pressure development, and metabolic function, and these features were maintained for 120 days in culture.^27^ Similarly, another study recently reported that human iPSC-CMs were repopulated in porcine decellularized myocardial slices.^28^ The engineered heart slices exhibited structural and functional improvements over 2D-cultured monolayers. In the construct, iPSC-CMs were organized into multicellular, aligned bundles with organized sarcomeres, and could maintain electrophysiological functionality over 200 days. When treated with ion channel-modulating drugs, the tissue slices showed different sensitives and could be electrically paced over a wider range of rates and drug concentrations than 2D-cultured monolayers, suggesting electrophysiological maturation. Wang *et al.* reported the regeneration of human cardiac patches by seeding human iPSC-CMs and human iPSC-CD90^+^ fibroblasts onto a decellularized heart ECM sheet.^29^ Upon transplantation, these patches improved the heart function of rats with acute myocardial infarction.^29^

The potential of decellularized organs as scaffolds has also been examined in other organ types. Previous studies reported that decellularized liver tissue retains key ECM components and growth factors to support hepatic differentiation and function of iPSCs.^30,31^ Supplementation of ECM from porcine decellularized liver tissue into culture medium enhanced albumin expression of porcine iPSC-derived hepatocytes (HEPs) during *in vitro* differentiation, at least partially due to the presence of growth factors in decellularized liver ECM.^30^ Then, the iPSC-HEPs were seeded into the decellularized rat liver tissues and perfused for 5 days. The recellularized liver was grafted by auxiliary heterotopic transplantation. The graft maintained its liver-specific functions and tolerated the blood pressure when filled with blood. Decellularized rat and human lung scaffolds have been demonstrated to be repopulated with iPSC-derived lung epithelial progenitor cells.^32,33^ For *in vitro* culture of recellularized lung grafts, bioreactors with continuous perfusion and ventilation system were applied to facilitate differentiation of iPSC-derived cells.^33,34^ Ren *et al.* reported methods for co-delivering iPSC-ECs and iPSC-perivascular cells to generate pulmonary vasculature in rat and human lungs.^34^ Using a customized reactor that allows cell delivery and perfusion, iPSC-ECs formed intact endothelial networks with iPSC-perivascular cells adhering around the vascular network. When the lung construct was grafted by orthotopic transplantation, the endothelium maintained continuous vascular lumen with intact barrier function for 3 days.^34^ Wan *et al.* demonstrated that mouse iPSC‐derived β‐like cells recellularized into decellularized pancreatic tissues showed enhanced cell survival and gene expression of insulin compared to a 2D-cultured monolayer.^35^ Humanized intestinal graft was tissue engineered by repopulating the lumen of decellularized intestine with human iPSC-derived intestinal epithelial progenitor cells and reconstructing the vasculature with human umbilical vein endothelial cells (HUVECs).^36^ After 2 weeks of static culture for stable epithelization, the construct was perfused to administer HUVECs through both arteries and veins and achieve endothelium. Accordingly, continuous intestinal epithelium with perfusable vasculature was formed in the construct. Four weeks after the recellularized intestine was transplanted to a subcutaneous pocket in the cervical region in a rat, the graft survival and epithelium regeneration were observed. Concerning the kidney, mouse or rat decellularized kidney was repopulated with human iPSC-derived renal progenitors and ECs.^37,38^ The presence of ECs increased the expression of renal-related genes, and when transplanted in mice, the formation of glomeruli was achieved in the graft.^36^

These studies have proven the potential of whole decellularized matrix as a natural scaffold platform for tissue engineering applications. However, only a few studies have investigated the regenerative performance of the recellularized tissue constructs *in vivo*. Furthermore, in-depth assessment and long-term observation of biocompatibility and functionality of whole decellularized matrices upon transplantation should be conducted in order to demonstrate the clinical feasibility for regenerative applications.

### C. Decellularized tissue-derived hydrogels

One issue associated with certain types of whole decellularized tissues is the difficulty in recellularization due to the complex architectures unfavorable for efficient cell seeding and uniform repopulation.^39^ To overcome this problem, decellularized tissues can be solubilized and then transformed into 2D surface coating materials and 3D hydrogels. Decellularized tissue-derived ECM solution can also be applied as a bioink for 3D printing.^40^ Recently, the decellularized matrix has been applied for improving direct reprogramming^41,42^ and iPSC differentiation.^43^ Jin *et al.* reported that 2D coating and 3D hydrogel platforms prepared from decellularized brain ECM facilitated the direct reprogramming of primary fibroblasts into induced neuronal (iN) cells.^41^ In particular, the 3D brain ECM hydrogel significantly promoted neuronal reprogramming and maturation via epigenetic modulation and mechanosensitive signaling pathways. Indeed, the 3D brain ECM hydrogel culture promoted phosphorylation of yes-associated protein and histone modification (H3K4me3 and H3K27ac). Generated iN cells showed remarkable therapeutic effects when transplanted in a mouse model with acute ischemic brain injury. Park *et al.* cultured human mesenchymal stem cells (MSCs) in the 3D-printed polycaprolactone (PCL) scaffolds soaked in decellularized heart ECM-derived bioink.^44^ They suggested the dual approach of human iPSC-CM injection and MSC-loaded PCL patch for treating myocardial infarction in a rat model. The epicardially transplanted patch could provide a complementary microenvironment by secreting paracrine factors, leading to the enhancement of vascular regeneration and the retention and engraftment of intramyocardially injected human iPSC-CMs.

### D. Synthetic scaffolds

Although natural polymer scaffolds have favored biocompatibility and innate biological signals, poor mechanical properties and the difficulty for modification may limit their applications. Synthetic polymers provide an artificial alternative with parameters tailored for specific applications including pore characteristics, degradation profiles, and mechanical properties. Several synthetic scaffolds, including poly(ethylene glycol) (PEG), PCL, and poly(lactide-co-glycolide) (PLGA), have been utilized for the expansion and differentiation of reprogrammed cells. In a previous study led by Ovadia *et al.*, degradable PEG-peptide-based hydrogels were used for iPSC culture and differentiation.^45^ The eight-arm PEG-norbornene was conjugated with a matrix metallopeptidase-degradable peptide sequence and integrin-binding motif to allow cell-driven remodeling and promote the binding of specific receptors on cells, respectively. The elasticity of the resultant hydrogels could be controlled by varying the amount of PEG. The authors found that both laminin-derived motif and integrin α_5_β_1_-binding motif resulted in the highest viability of iPSCs and differentiation into NPCs. Cho *et al.* utilized aligned electrospun nanofibrous PCL scaffolds for co-culture of directly reprogrammed iN cells and human iPSC-derived oligodendrocytes (OLs).^43^ The scaffolds were functionalized with the decellularized brain ECM to reconstitute brain-like biochemical, biophysical, and structural signals for promoting the maturation of iPSC-OLs.^43^ The co-culture of iN cells and iPSC-OLs not only enhanced the formation of myelin sheath-like structures of iPSC-OLs, but also enhanced the neurogenesis of iN cells. Lancaster *et al.* used PLGA fiber microfilaments as an internal scaffold to improve the architecture of the brain organoids by elongating the embryoid body (EB) from the inside.^46^ Micrometer-scale filaments contacted only the innermost layer of cells of the EB enhanced neuroectoderm formation with larger and continuous cortical lobes and improved cortical development.

Synthetic scaffolds have also been used to improve the cell delivery efficiency upon transplantation. Delivery of iPSC-ECs with shear-thinning hydrogel injection improved acute viability.^47^ The hydrogel was prepared with C7 engineered recombinant protein and a multiarmed, P1-peptide-modified PEG with thermoresponsive poly(N-isopropylacrylamide) (PNIPAM). The shear-thinning hydrogel protected cells from the shear force during injection through syringe, and secondary *in situ* cross-linking improved cell retention *in vivo*. In a murine hindlimb ischemia model, transplantation of iPSC-ECs using the hydrogel improved neovascularization in the ischemic limb tissue via arteriogenesis. Yang *et al.* demonstrated that iPSC-derived neural stem cell (NSC) delivery with biodegradable hybrid inorganic nanoscaffolds facilitated transplantation of stem cells into spinal cord injury sites.^48^ The scaffolds were self-assembled from MnO_2_ nanosheets, ECM proteins (laminin), and neurogenic drugs. MnO_2_ nanosheets have stronger binding affinity to laminin, which improved adhesion, axonal growth, and differentiation of iPSC-NSCs. Neurogenic drug-loaded scaffolds further enhanced neuronal differentiation, while suppressing glial differentiation. Transplantation of iPSC-NSCs with this scaffold improved cell survival, induced neuronal differentiation, and reduced glial scar formation in a murine spinal cord injury model, compared to the treatments with cells using control materials (PCL or laminin).

## III. 3D BIOPRINTING

Bioprinting guides the assembling process of a 3D construct, as it enables precise control over spatial distributions of cells and multiple compositions of matrices at the micrometric scale. The advent in 3D bioprinting holds a promise for artificial tissue fabrication for transplantation, disease modeling, and drug testing. Despite these advantages, application of 3D bioprinting to reprogrammed cells is still in its infancy.^49^ Only very recently, iPSCs and iPSC-derived cells alone or with scaffolds have been used in combination with 3D bioprinting techniques to fabricate the constructs of desired architectures. Undifferentiated iPSCs could first be printed and then induced to differentiate into target cells, or lineage-specified cells derived from iPSC could be printed. In the former, EB formation and direct differentiation of human iPSCs within the printed construct have been demonstrated, along with cartilage,^50^ cardiac,^51^ and neural^52^ lineages. The reversal approach of bioprinting lineage-specified cells in the latter has been reported with CMs,^53,54^ HEPs,^53,55^ ECs,^56^ and neural cells.^57,58^ However, in-depth investigation on regenerative effects of the 3D-bioprinted constructs has rarely been reported. Moreover, application of bioprinting technology has not been reported for directly reprogrammed cells.

Various types of bioprinting techniques, including valve-based,^55^ extrusion,^50,52,59,60^ and laser-assisted^51^ printing, have been applied to print undifferentiated iPSCs for scalable cell expansion. The key interest in bioprinting is to maintain or enhance the self-renewal ability of iPSCs and their capability to differentiate into specific cell types. With a set of the optimized parameters, maintenance of cell survival and higher proliferation after the printing process was achieved by several bioinks including hyaluronic acid,^51^ carboxymethyl-chitosan,^52^ agarose,^52^ cellulose,^50^ alginate,^55^ gelatin,^61^ and hydroxypropyl chitin.^60^ Laser-printing of undifferentiated iPSCs with various types of biomaterials was conducted.^51^ Of the tested conditions, which included collagen, alginate, and hyaluronic acid, superior survival and reproducibility were achieved when printing with combination of culture medium and hyaluronic acid on the substrate coated with Matrigel. In other study, iPSCs were co-printed with irradiated human chondrocytes in a bioink composed of nanofibrillated cellulose (NFC) to form cartilaginous tissue.^50^ When the cells were printed with NFC bioink with hyaluronic acid, lower proliferation of the iPSCs was observed, whereas NFC with alginate supported the pluripotency of the cells. Furthermore, enhanced cartilage differentiation was observed in 3D-bioprinted NFC with alginate construct. It was found that the cells were more sensitive to the applied biomaterials but not to printing process itself.

### A. Heart

Bioprinting techniques have most extensively been employed for cardiac construction. Both scaffold-free and scaffold-based techniques have been applied to fabricate 3D cardiac-engineered constructs. Among the scaffold-free 3D printing techniques, cell spheroids consisting of iPSC-CMs, HUVECs, and human dermal fibroblasts were placed on a needle array creating a large tubular cardiac construct^62^ or a cardiac patch.^54^ These 3D bioprinted constructs formed with spheroids showed the potential to develop functionalized large cardiac constructs. In the cases of scaffold-based 3D constructs, a majority of studies fabricated scaffolds before adding iPSC-CMs. Gao *et al.* used a high-resolution multiphoton-excited 3D printing to generate a scaffold with native heart-like ECM architecture using photoactive gelatin polymer and a template prepared on the basis of the distribution of fibronectin in the murine myocardium.^63^ The gelatin scaffold was then cultured with CMs, SMCs, and ECs differentiated from human cardiac fibroblast-derived iPSCs to generate an engineered heart muscle (EHM) patch. Synchronous beating and continuous action potential propagation in the construct were observed during extended *in vitro* culture over 7 days. The transplantation of the cardiac patch into immunodeficient mice with myocardial infarction improved cardiac function.^63^ Another study described the development of an EHM by preprinting flexible circular casting molds and culturing cardiac fibroblasts and iPSC-CMs on the molds.^64^ EHM responded to cardiotoxic substances indicating hallmarks of heart failure.

In an effort to induce vascularization, Maiullari *et al.* developed a microfluidic head-bioprinting method to simultaneously extrude multiple bioinks and control spatial deposition at a high resolution.^65^ HUVECs and iPSC-CMs in alginate and PEG-fibrinogen were extruded. The outlet channel was fluidically connected to a co-axial nozzle extruder so that the bioinks and calcium chloride solution could be simultaneously co-extruded as fibers. Then, the construct was treated with ultraviolet in which the vinyl moieties of PEG-fibrinogen were polymerized. In the generated cardiac tissues, iPSC-CMs showed a high orientation index imposed by the different defined geometries and blood vessel-like structures generated by HUVECs were also observed. A recent paper from Noor *et al.* reported a 3D model of personalized vascularized cardiac patches by printing a bioink in supplementary material.^66^ A biopsy of an omental issue was harvested for both extracting omental stromal cells and preparing decellularized ECM bioink. The stromal cells were then reprogrammed to iPSCs and subsequently differentiated into either CMs or ECs. The construct was fabricated by free‐form printing as the heart-like structure inside the support bath consisting of alginate microparticles in growth medium supplemented with xanthan gum.^66^ The bioink was then crosslinked to form a hydrogel by incubation at 37 °C, and the support material was removed by an enzymatic or chemical degradation. This study demonstrated that the construct can be fully personalized by using iPSC-derived cells and decellularized tissue matrices from the same patient. More recently, patterning of iPSC-cardiac spheroids was demonstrated by bioprinting spheroids inside support hydrogel.^67^ The support hydrogel was composed of hyaluronic acid modified with adamantane or β-cyclodextrin that has shear-thinning and self-healing characteristics. The spheroids were bioprinted with direct contact and these spheroids fused to form a microtissue. The focal cardiac fibrosis was modeled by depositing normal spheroids and scarred spheroids generated by changing the ratios of iPSC-CMs and cardiac fibroblasts. The resultant model not only showed structural and functional resemblance of scarred cardiac tissue, but also validated improvement in electrophysiological properties by microRNA treatment.

### B. Liver

The liver tissue is comprised of HEPs assembled in 3D hexagonal lobule units and surrounded by supporting cells. Since this characteristic microstructure organization is critical for hepatic function, bioprinting technology has been applied to generate liver tissue constructs with structural similarity to the *in vivo* counterparts. Ma *et al.* reported the sequential printing of human iPSC-HEPs in a honeycomb pattern of hexagons, with supporting ECs and mesenchymal cells that fill the spaces between the hexagonal lobule structure.^68^ A layer of human iPSC-HEPs in photo-crosslinkable gelatin methacrylate (GelMA) was assembled with a layer of supporting cells in the mixture of GelMA and glycidyl methacrylate-hyaluronic acid using digital light processing (DLP)-based 3D bioprinting system.^68^ The 3D printed liver construct showed prolonged cell viability and enhanced hepatic functionality compared with 2D monolayer culture and 3D model comprised of only human iPSC-HEPs. More recently, Yu *et al.* reported application of photo-crosslinkable tissue-specific ECM bioinks and 3D DLP printing technique to generate cardiac and liver tissues.^53^ In a combination of decellularized tissue-derived ECM solution and GelMA, the mechanical properties of the bioink could be controlled by varying the cross-linking density via polymerization of the GelMA, while retaining tissue-matched biological components. The bioink was printed with iPSC-CMs and iPSC-HEPs to recapitulate striated heart and lobular liver microarchitectures, respectively, and the resultant constructs showed high viability and maturation of the printed cells.

### C. Nervous system

In order to replicate complex neural structures, precise control of the organization of cells and the microenvironment complexity are crucial. Bioprinting approaches have been employed to find viable solutions.^57^ For example, Joung *et al.* developed a bioengineered spinal cord by bioprinting iPSC-derived oligodendrocyte progenitor cells (OPCs) and spinal NPCs in alternating positions in 3D microchannels.^58^ The OPCs or spinal NPCs were alternatively deposited as clusters resuspended in Matrigel solution by point-dispensing printing method. An extensive outgrowth of axons within the scaffold was observed. Another study demonstrated extrusion printing of human iPSCs and hydrogel solutions (alginate, carboxymethyl-chitosan, and agarose) *in situ* to induce EB formation and subsequent differentiation into neuronal and glial lineage cells.^52^

### D. Skin

Materials commonly used for skin bioprinting include natural materials, such as chitosan, hyaluronic acid, collagen, gelatin, and fibrin and synthetic materials, such as polylactic acid (PLA), PCL, PEG, or a combination of PEG-diacrylate and GelMA.^69^ Perfusable 3D vascular networks were incorporated into the skin construct by utilizing bioprinting technology. Abaci *et al.* fabricated a 3D skin construct with vasculature by seeding iPSC-ECs in the 3D-printed mold.^70^ The 3D-printed molds with vasculature pattern was used to create a sacrificial layer of alginate microchannels, which were subsequently embedded with dermal fibroblasts and collagen gel forming the dermal compartment.^70^ The keratinocytes were then seeded on top of the dermal compartment and underwent epidermalization. At this point, the sacrificial layer was dissolved by adding sodium citrate and followed by iPSC-EC seeding in the microchannels.

## IV. MICRODEVICE PLATFORMS

**FIG. 2.**
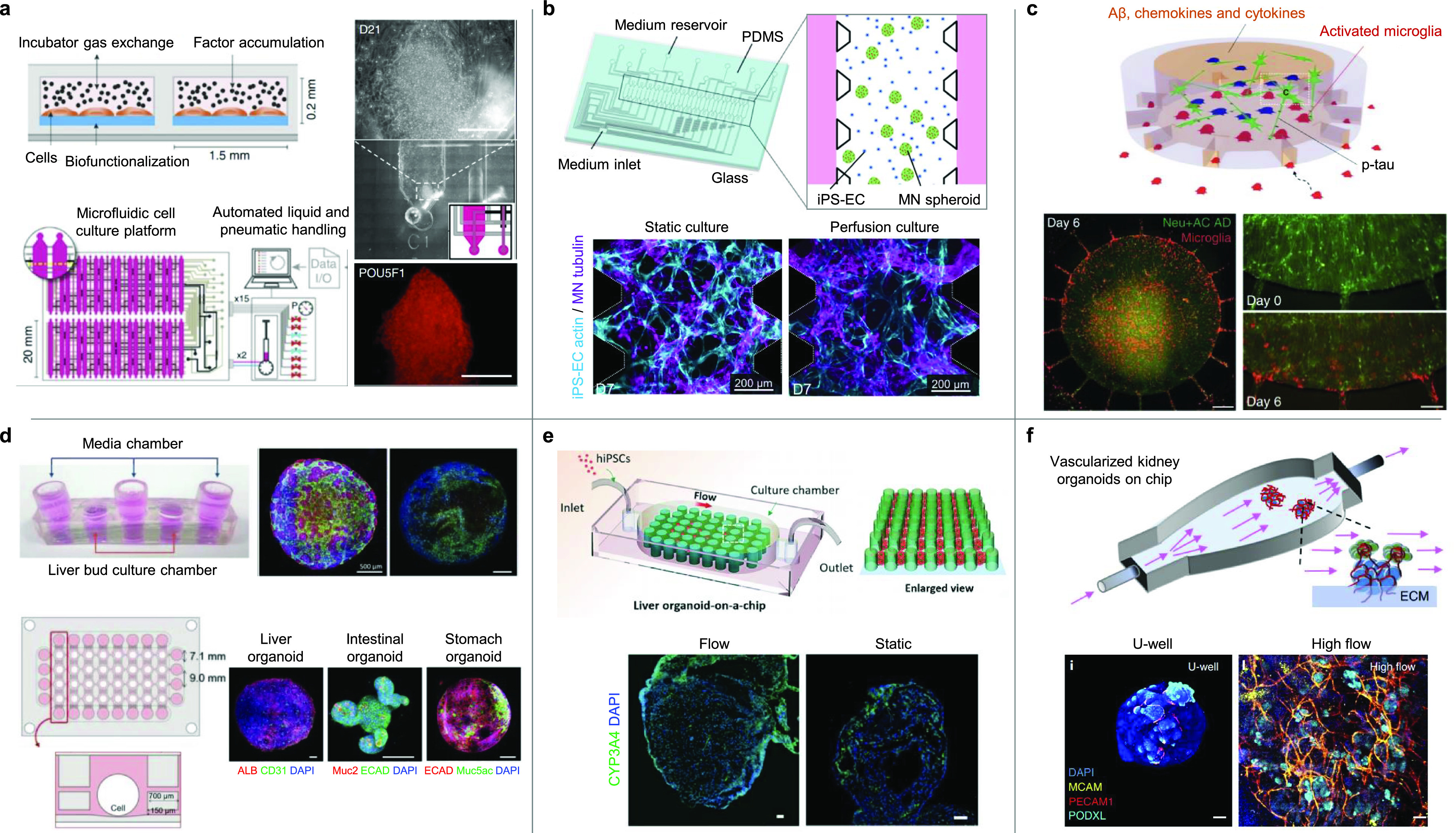
Diverse applications of microdevice platforms in induced pluripotent and direct reprogramming and differentiation. (a) Generation of human iPSCs at a higher efficiency of reprogramming in the microfluidic cell culture system.^72^ Reprinted by permission from Luni *et al.*, Nat. Methods **13**(5), 446–452 (2016).^72^ Copyright 2016 Springer Nature Customer Service Center GmbH: Springer Nature. (b) Formation of motor neuronal and vascular networks in a multichannel microfluidic device in the presence of perfusion culture.^77^ Adapted with permission from Osaki *et al.*, Sci. Rep. **8**, 5168 (2018). Copyright 2018 Authors, licensed under a Creative Commons Attribution (CC BY) license. (c) A 3D organotypic Alzheimer's disease (AD) model by triculture of AD neurons, astrocytes, and microglia in a 3D microfluidic platform.^78^ Reprinted by permission from Park *et al.*, Nat. Neurosci. **21**(7), 941–951 (2018).^78^ Copyright 2018 Springer Nature Customer Service Center GmbH: Springer Nature. (d) Construction of 3D vascularized induced hepatic tissues generated in a 3D microfluidic system under flow conditions.^42^ Generation of a multiorgan model by tri-culturing 3D hepatic tissues, intestinal organoids, and stomach organoids in a high‐throughput microfluidic device. Adapted with permission from Jin *et al.*, Adv. Funct. Mater. **28**, 1801954 (2018). Copyright 2018 Wiley‐VCH Verlag GmbH & Co. KGaA, Weinheim. (e) *In situ* generation of liver organoids using a 3D perfusable chip.^83^ Republished with permission from Wang *et al.,* Lab Chip **18**, 23 (2018). Copyright 2018 Clearance Center, Inc. (f) Development of vascularized and mature renal organoids by placing them on ECM within a perfusable millifluidic chip.^81^ Reprinted by permission from Homan *et al.*, Nat. Methods **16**(3), 255–262 (2019).^81^ Copyright 2019 Springer Nature Customer Service Center GmbH: Springer Nature.

Recent advances in microfabrication platforms (e.g., microfluidics) have provided improved methods of mimicking the complexity of tissue architectures. Microfluidic systems provide an efficient method to control biophysical microenvironmental conditions. In addition, autocrine and paracrine factors are highly concentrated in the microfluidic system, or the gradients of such factors for *in vivo*-like biochemical signals can be reconstituted. Thus, microfluidics has been successfully implemented to manipulate cell fate control.^71–73^ With these advantages of microfluidic systems, they have been widely applied for pluripotent reprogramming, direct conversion, and organoid development (Fig. 2).

### A. Microfluidic devices for reprogramming

Human iPSCs have been generated by vector-free gene delivery using microfluidic chips.^71,74^ Cells were subjected to a controlled mechanical deformation by passing through a microfluidic channel smaller than cell diameter.^74^ The application of rapid compression and shear forces resulted in the formation of transient holes in cell membranes, leading to intracellular transfer of materials from the surrounding medium. Direct-to-cytosol delivery of transcription factors by this system resulted in the ten-times higher efficiency of pluripotent reprogramming than the conventional methods using electroporation and cell-penetrating peptides. Another study demonstrated a method to generate human naïve iPSCs directly from fewer than 1000 primary fibroblasts by delivering modified messenger RNAs in the microfluidics.^71^ Significantly higher efficiency of iPSC colony formation was achieved with this system. The other study also reported that the efficiency of pluripotent reprogramming was improved in a microfluidic environment.^72^ Microvolume confinement within the device resulted in a dramatic increase in iPSC generation efficiency. The resulting iPSCs were able to directly differentiate into functional HEPs and CMs in the same microfluidic platform without additional expansion. The same research group demonstrated a microfluidic-based strategy for direct differentiation of human iPSCs on a chip.^75^ Germ layer specification and differentiation were improved by modulating extrinsic signals, which was achieved with optimal frequency of medium change in the microfluidic device. iPSC-CMs and HEPs directly differentiated on chips exhibited functional phenotypes and defined responses to drug treatment.

Flow-focusing microfluidics was applied to generate microbeads for 3D culture and transplantation of reprogrammed cells.^76^ Decellularized tissue-derived ECM solutions were used to encapsulate induced hepatic, cardiac, and myogenic cells all directly reprogrammed from fibroblasts in a tissue type-matched manner.^76^ The tissue-specific ECM microbeads supported higher cellular viability and improved maturation and functionality, compared with the collagen type I microbeads. The microbeads mediated successful *in vivo* engraftment of reprogrammed hepatic or myogenic cells in injured tissue, compared with the conventional methods by injection of cells using culture medium or collagen beads.

### B. Organ-on-a-chip platforms

Organ-on-a-chips combine microfluidics with tissue engineering concept to serve as 3D culture platforms that mimic the native organ environment. In microfluidic organ-on-a-chip models, structural and functional features of native organs can be recreated through fine control of experimental variables including fluid flow, cell–cell interactions, matrix properties, and biochemical and biomechanical cues. Osaki *et al.* developed a 3D perfusable vasculature and neuronal network model in a multichannel microfluidic platform using human embryonic stem cell (ESC)-derived motor neurons and iPSC-derived ECs.^77^ The perfusable microvascular network influenced synaptic connectivity via direct and indirect signaling, while motor neuron network also promoted vascular network formation. More recently, a 3D multichambered microfluidic device was used to tri-culture cells (iPSC-derived neurons and astrocytes, and immortalized microglia) to model Alzheimer's disease (AD).^78^ The 3D tri-culture system modeled more physiologically relevant neural–glial interactions and pathogenic cascades of the disease. This system enabled formation of the gradients of inflammatory factors secreted from AD neurons and astrocytes that promote microglia recruitment toward neurons and astrocytes. Recruited microglia induced damages to AD neurons and astrocytes so that they expressed higher levels of pathological features. Our group employed decellularized liver-derived ECM and microfluidic chips to improve the generation of vascularized hepatic tissues.^42^ Directly reprogrammed hepatic-like cells and ECs were co-cultured in decellularized liver ECM hydrogel under a dynamic fluid flow. Vascularized liver tissues that possess mature hepatic functions were produced via reconstituted dynamic liver-mimetic microenvironment in microfluidic devices. Not only was the feasibility for high‐throughput drug screening proven, but also multiorgan model integrated with other organoids such as intestine and stomach was demonstrated.

Recently, the organ-on-a-chip has been used for iPSC-derived organoids.^79–81^ The organ-on-a-chip approaches enable the supply of controlled fluidic conditions that support the growth of organoids to the millimeter size via improved distribution of nutrients and oxygen. For example, Wang *et al.* developed a brain organoid-on-a-chip model in microfluidics.^79^ Under the perfused flow, iPSC-derived brain organoids exhibited an increased expression of cortical layer markers, compared with those grown under static culture in a Petri dish. The follow-up study performed by Wang *et al.* applied the same microfluidic configuration to investigate the effects of prenatal nicotine exposure on brain organoids.^82^ Upon exposure to nicotine, microfluidic brain organoids underwent premature neurogenesis and abnormal growth of brain regions.

There have been more examples to employ microfabricated systems for improving iPSC-derived organoid culture. Wang *et al.* fabricated a microfluidic device with micropillar array structures for the production of human iPSC-derived EBs and *in situ* generation of liver organoids.^83^ Under the perfusion conditions, cells dispersed throughout the channels between the pillars, formed unformed EBs, and consequently liver organoids. Similarly, the same group reported fabrication of human iPSC-derived islet organoids in a multilayer microfluidic device that can permit circulatory flow, which facilitates efficient medium exchange and uniform fluid stress to the organoids.^84^ The microfluidic platform allowed controllable formation of EBs, *in situ* pancreatic differentiation, and maturation of islet organoids. Synergistic engineering strategy has also been proposed to integrate iPSC-renal organoids, engineered ECM, and the millifluidic device for the generation of flow-mediated vascularized and mature kidney organoids.^81^ The fluid flow expanded the population of endothelial progenitor cells that generate vascular networks with perfusable lumens, and more mature podocytes and tubular compartments. A series of these studies showed how microfluidics can address the issue of uncontrolled microenvironment in conventional organoid culture systems.

Scalability is one of the largest hurdles in reprogramming and organoid technology. Toward this end, Takebe *et al.* built a large-scale liver bud microwell culture platform (20 000 microwells/plate in omni-plate format) enabling a clinically relevant mass production.^85^ Vascularized liver buds generated entirely from iPSCs in the microwell culture platform exhibited significantly improved hepatic functionalization and functional rescue against acute liver failure upon transplantation. Scale-up production of EBs was demonstrated using the micropillar array chip, which enables generation of a large number of EBs with controlled size and direct development of EBs into brain organoids.^86^ Spinning bioreactors have been employed to achieve massive production of iPSC-macrophages^87^ and iPSC-brain organoids.^88^

## V. BIOSTIMULATION

**FIG. 3.**
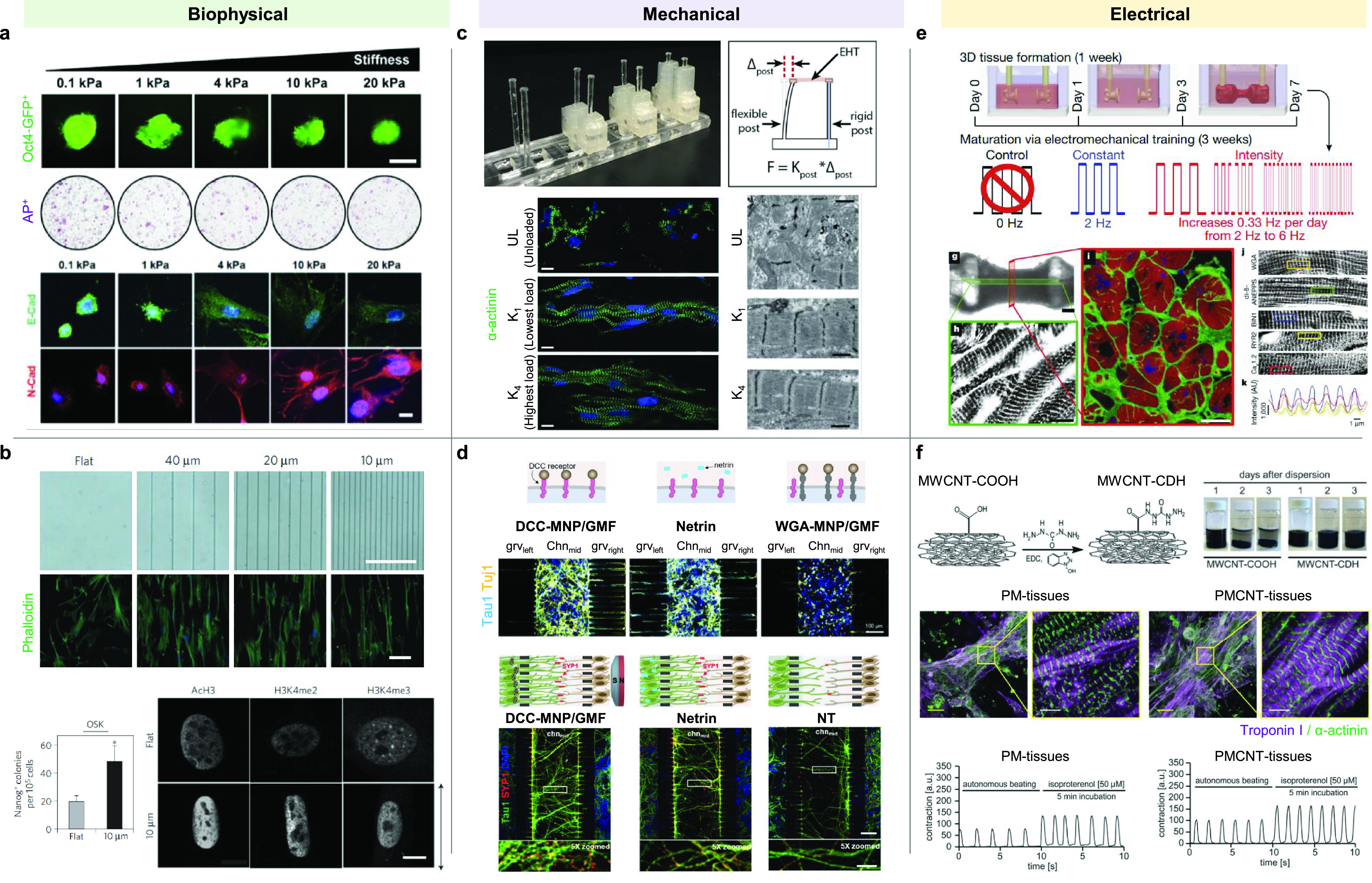
Biostimulatory platforms to promote induced pluripotent and direct reprogramming and maturation. (a) Stiffness of hydrogels affects mesenchymal–epithelial transition and reprogramming efficiencies of generating iPSCs.^89^ Adapted with permission from Choi *et al.*, Macromol. Biosci. **16**, 199 (2015). Copyright 2015 Wiley-VCH Verlag GmbH & Co. KGaA, Weinheim. (b) Microgroove patterned substrates affect fibroblast morphology and iPSC generation.^97^ Reprinted by permission from Downing *et al.*, Nat. Mater. **12**(12), 1154–1162 (2013).^97^ Copyright 2013 Springer Nature Customer Service Center GmbH: Springer Nature. (c) Afterload promotes maturation of engineered heart muscles.^101^ Reprinted with permission from Leonard *et al.*, J. Mol. Cell. Cardiol. **118**, 147–158 (2018). Copyright 2018 Elsevier. (d) Control of axonal growth of iN cells by receptor conjugated-magnetic nanoparticles (MNP) and gradient magnetic field (GMF).^102^ Adapted with permission from Jin *et al.*, Nano Lett. **19**, 6517 (2019). Copyright 2019 American Chemical Society. (e) Gradual increase in frequency during electrical stimulation of iPSC-CMs encapsulated in fibrin hydrogel promotes cardiac maturation.^106^ Reprinted by permission from Ronaldson-Bouchard *et al.*, Nature **556**(7700), 239–243 (2018).^106^ Copyright 2018 Springer Nature Customer Service Center GmbH: Springer Nature. (f) Hydrazide-functionalized carbon nanotube-pericardial matrix (PMCNT)-derived conductive hydrogel enhances cardiac maturation.^110^ Republished with permission from Roshanbinfar *et al.*, Biomater. Sci. **7**, 9 (2019). Copyright 2019 Clearance Center, Inc.

Cells in their native *in vivo* environments experience topographical and biophysical cues, mechanical cues, and electrical signals. For example, vascular and cardiac cells experience constant physiological mechanical forces, shear from blood flow, and rhythmic contractions. These dynamic environments are often coupled with ECM and have both cell intrinsic and extrinsic effects. There have been several studies aiming to recapitulate such forces and signals during programming and subsequent differentiation for developing the engineered constructs with increased similarity to their native counterparts (Fig. 3).

### A. Biophysical cues

Biophysical cues, which include stiffness and topography, can be varied to manipulate cell fate. A series of studies have shown that stiffness is one of the key parameters to consider in iPSC reprogramming^89,90^ and differentiation strategies.^91^ For example, neural differentiation of human iPSCs in methacrylated hyaluronic acid hydrogel was promoted under conditions of lower stiffness, whereas higher hydrogel stiffness was capable of better maintaining the progenitor properties of human iPSC-derived NPCs.^19^

Topography can also play an important role in guiding cell fate. Materials with different topographical cues have been developed through a variety of methods including electrospinning,^92,93^ pore shape manipulation,^94^ lithography,^95^ and surface treatments.^96^ The effect of biophysical cues on iPSC reprogramming was first demonstrated by Downing *et al.* showing that microtopography in the form of microgrooved surfaces could replace the roles of small molecules for epigenetic modification.^97^ Accordingly, the application of microgroove patterned substrates improved reprogramming efficiency via modulating the epigenetic state. Aligned nanofibrous scaffolds induced the effects similar to microgroove patterns, suggesting that changes in cellular morphology may be responsible for epigenetic modification.^97^ The 3D geometrical confinement of human iPSC colonies on PEG-patterned substrates modulated spatial mechanical stress and induced early lineage specification, leading to generation of a beating human cardiac microtissue.^98^ In another study, the patterning of iPSC-CMs in 3D-rectangular-shaped scaffolds was investigated.^99^ As compared with nonpatterned cells, the 3D-shaped iPSC-CMs showed reorganization of Ca^2+^ handling proteins, enhanced spontaneous Ca^2+^ transients, and Ca^2+^ handling, suggesting that structural remodeling improved structural organization and electrophysiological properties of cells.

### B. Mechanical cues

In the mechanical aspect, the application of stretch or afterload on 3D EHM tissues with human iPSC-CMs promoted their structural and functional maturation.^100,101^ A passive stretch facilitated metabolic switches in human iPSC-CMs.^13^ The work performed by Abilez *et al.* showed that passive stretching of EHM improves its maturity.^100^ The EHM constructs were generated by mixing human iPSC-CMs or human ESC-CMs in collagen hydrogels. EHM was grown between two polydimethylsiloxane (PDMS) posts of varying distances, and the EHM was stretched to various lengths from 0 (with one post only) to 9 mm. The resulting EHM displayed significant variations in calcium handling under different stretching conditions. For instance, tension with 7 mm distance led to the highest calcium amplitude and slower beating rate and longer duration, suggesting that the modulation of tissue stress has a significant effect on its functional performance. The EHMs also exhibited higher expression of genes related to maturation than the cells grown as monolayers.

Artificial biochemical guidance by magnetic field-induced mechanical cues has been applied for controlling axonal projection of directly reprogrammed iN cells and human iPSC-NPCs.^102^ Our group devised a magnetic guidance strategy that enables spatial control of axonal growth via magnetic nanoparticles (MNPs) conjugated with the deleted colorectal cancer (DCC) receptor-targeting antibodies. The MNP-bound DCC receptors on iN cells were clustered toward the gradient magnetic field, leading to the spatially controlled axonal projection of the iN cells. Furthermore, axonal growth of iN cells assisted the formation of synaptic junctions with neighboring primary neurons. This magnetic guidance would be applied for spatially controlling axonal direction of transplanted iN cells to form neuronal networks with the host neurons, thereby improving therapeutic efficacy of iN cells.

### C. Electrical stimulation

The body generates endogenous electric fields that affect many important biological processes, such as mitosis, migration, and wound healing.^103^ Indeed, several studies have shown that exogenous electrical stimulation guides a variety of cellular behaviors, including proliferation, differentiation, and maturation *in vitro*. Electrical cue is particularly influential for neuronal cells^21,104^ and cardiac cells.^105,106^ For instance, our group investigated the effects of electrical stimulation on the direct reprogramming of fibroblasts to neuronal cells.^21,104^ A self-powered triboelectrical nanogenerator was applied as an electrical stimulation source to generate biphasic pulse‐like currents to promote direct neuronal reprogramming. The electrical stimulation, combined with the nonviral transfection of neuronal lineage-related transcription factors, accelerated neuronal reprogramming with increased efficiency both *in vitro* and *in vitro*. Shin *et al.* developed an electrically conductive hydrogel by incorporating single-walled carbon nanotubes and polypyrrole nanocomposites into catechol-functionalized hyaluronic acid hydrogel for neuronal differentiation.^21^ Electrically conductive hydrogels promoted the differentiation of human iPSC-NPCs and improved their electrophysiological functionality, as compared with the cells cultured in a hydrogel without electroconductive nanomaterials. Recently, Tsui *et al.* developed a hybrid hydrogel composed of decellularized myocardial ECM and reduced graphene oxide.^107^ The mechanical and electrical properties of the hydrogel could be tuned. The engineered cardiac tissue with iPSC-CMs in this hydrogel system showed enhanced contractile function and improved electrophysiological function. Importantly, the resultant cardiac construct displayed physiologically relevant drug responses at clinical doses. Electromagnetized gold nanoparticles under electromagnetic fields (EMFs) enhanced direct reprogramming of fibroblasts into dopaminergic neurons.^108^ Application of electromagnetic stimulation to the fibroblasts transfected with dopaminergic neuron-specific transcriptional factors led to a specific activation of the histone acetyltransferase Brd2, which in turn induced histone H3K27 acetylation and subsequent activation of neuronal genes. *In vivo* dopaminergic neuronal reprogramming by transcription factor delivery and the EMF stimulation of gold nanoparticles restored neurodegenerative symptoms in a mouse model of Parkinson's disease.

Electrical stimulation has also been employed to improve the maturity of CMs.^105,106^ Human iPSC-CMs in a collagen gel were embedded into a PDMS channel to form a wire-like structure. Upon exposure of electrical stimuli, functionally more mature cardiac tissue was generated.^105^ In other studies, electroconductive biomaterials have been employed for human iPSC-CM maturation.^109,110^ For example, silicon nanowires were incorporated into CM spheroids to form an electrically conductive environment. Combination of silicon nanowire and exogenous electrical stimulation further improved functional maturation of CM spheroids.^111^ In another study, carbon nanotubes were added in decellularized pericardial matrix.^110^ Human iPSC-CMs cultured in this conductive ECM hydrogel exhibited improved cellular alignment, sarcomeric organization, and calcium handling compared to the cells cultured in Matrigel. More recently, maturation of chemically induced CMs was demonstrated by using a micropillar electrode array.^112^ Fibroblasts in a microwell culture were treated with a small-molecule cocktail to generate induced CM spheroids. Direct cardiac reprogramming was enhanced by 3D spheroid culture, compared with the 2D monolayer culture. The biphasic electrical stimulation into induced CM spheroids via a micropillar electrode array further improved maturation and electrophysiological properties of the spheroids, leading to higher sensitivity to drugs than the spheroids without the treatment of electrical pulses.^112^

More sophisticated stimulation has been tested by several groups to generate EHMs. EHMs assembled from early stage CMs were exposed to a high-intensity training regimen of biphasic electrical pulses with gradually increasing the frequency from 2 to 6 Hz. The resulting cardiac tissue displayed physiologically adult heat-like features, including well-organized ultrastructure, oxidative metabolism, and calcium handling.^106^ The combinations of electrical and mechanical stimulation (e.g., cyclic stretch and static stress) have also been explored.^113,114^ In such conditions, human iPSC-CMs displayed more mature cardiac signatures as compared to when treated with a single stimulus.

## VI. CONCLUSIONS

Tremendous progress has been recently made in the use of bioengineering tools to improve pluripotent and directly reprogrammed cell therapeutics. Bioengineering technologies, such as biomaterial scaffolds, bioprinting, microfabricated devices, and biostimulatory systems, may help circumvent some of the hurdles associated with current iPSC and reprogramming techniques and enable the establishment of more stable and effective procedures to facilitate clinical translation of iPSC-derived and directly reprogrammed cell therapy. However, there are several limitations with these technologies at current state. Most recellularized organs and bioprinted constructs require bioreactors to sustain tissue viability during maturation period. A new engineering approach should be considered to provide *in vivo*-like environment allowing for long-term cultivation of the engineered tissues. Another challenge of bioprinting is to create highly organized, perfusable vascular networks within the printed constructs, which has been impeded by low resolution and slow speed of current printing techniques. For biostimulatory approaches to improve reprogramming efficiency and target lineage differentiation, underlying mechanisms and modes of action have not been extensively investigated yet. We have also encountered several major issues that need to be solved in order to integrate these technologies into clinical practices. The safety of cell reprogramming is a major outstanding challenge which needs to be verified in long-term clinical situations. Other issues of batch-to-batch variation, treatment convenience, and time frame of cell generation should also be considered together. Although the bioengineering technologies have been outlined individually, distinct technologies have emerged into one platform through synergistic integration in recent years. For example, a combination of tissue ECM engineering and microfluidic system successfully constructed highly vascularized organoids with improved maturity.^42,81^ Such integrative innovation can mitigate limitations of each approach and provide synergistic combination of each technological advantage, finally leading to the next generation of reprogrammed cell-based regenerative medicine for transplantation, disease modeling, and drug screening.

## Data Availability

Data sharing is not applicable to this article as no new data were created or analyzed in this study.
